# PD-L1 Expression in High-Risk Non-Muscle-Invasive Bladder Cancer Is Influenced by Intravesical Bacillus Calmette–Guérin (BCG) Therapy

**DOI:** 10.3390/cancers16071356

**Published:** 2024-03-29

**Authors:** Moritz Maas, Andreas Hilsendecker, Alexandra Pertoll, Viktoria Stühler, Simon Walz, Steffen Rausch, Arnulf Stenzl, Igor Tsaur, Jörg Hennenlotter, Stefan Aufderklamm

**Affiliations:** 1 Department of Urology, University Hospital Tübingen, Eberhard Karls University, 72076 Tuebingen, Germany; andreas.hilsendecker@student.uni-tuebingen.de (A.H.); alexandra.pertoll@student.uni-tuebingen.de (A.P.); viktoria.stuehler@med.uni-tuebingen.de (V.S.); simon.walz@med.uni-tuebingen.de (S.W.); steffen.rausch@med.uni-tuebingen.de (S.R.); eau@stenzl.net (A.S.); igor.tsaur@med.uni-tuebingen.de (I.T.); joerg.hennenlotter@med.uni-tuebingen.de (J.H.); stefan.aufderklamm@med.uni-tuebingen.de (S.A.); 2 Department of Urologic Sciences, University of British Columbia, Vancouver, BC V5Z 1M9, Canada; 3 Department of Urology, General Hospital of Bregenz, 6900 Bregenz, Austria

**Keywords:** bladder cancer, non-muscle-invasive bladder cancer, immunotherapy, BCG, biomarker

## Abstract

**Simple Summary:**

While immunotherapy with checkpoint inhibitors is a standard component of treatment for advanced bladder cancer, its potential in early-stage, non-muscle-invasive bladder cancer (NMIBC) is increasingly being evaluated. Traditionally, NMIBC is managed with Bacillus Calmette–Guérin (BCG), a therapy that activates the immune system. Given that PD-L1 protein expression is an important marker for predicting the response to immunotherapy in advanced stages, and has shown prognostic value, and considering that BCG therapy functions by stimulating the immune system, our aim is to investigate whether PD-L1 levels change over time or with BCG treatment in high-risk NMIBC patients, and the prognostic implications thereof. This research could offer new insights into biomarker expression in early-stage bladder cancer by evaluating its susceptibility to therapies. The capacity of BCG to influence PD-L1 expression might provide hints for a sequential application of therapies.

**Abstract:**

In the expanding landscape of immune checkpoint inhibitors (CPI) in high-risk (HR) non-muscle-invasive bladder cancer (NMIBC), the role of programmed death ligand 1 (PD-L1) as prognostic and predictive is increasingly significant. However, data evaluating its variability and susceptibility to Bacillus Calmette–Guérin (BCG) therapy in HR NMIBC patients is scarce. This retrospective study analyzed 126 HR NMIBC tissue samples from 63 patients (38× BCG-treated, 25× BCG-naïve) at two time points to assess PD-L1 expression using the ‘combined positivity score’ (CPS) with the 22C3 DAKO antibody method and correlated it with clinicopathological parameters. A CPS > 10 defined PD-L1 positivity. The impact of initial PD-L1 status and its change over time on time-to-recurrence, progression-free survival, and overall survival (TTR, PFS, OS) was analyzed using Kaplan–Meier and Cox proportional hazard models. BCG treatment significantly increased PD-L1 expression (5.31 vs. 0.22, *p* = 0.0423), with PD-L1 positive cases rising post-treatment in the BCG group and remaining unchanged in BCG-naïve patients. Multivariate analysis including T-stage, CIS, grading, tumor size, multifocality, age, and sex revealed a significant correlation between PD-L1 status change to positivity and improved TTR (*p* = 0.03). Our findings demonstrate a potential modulation of the PD-L1 status by an intravesical BCG therapy. However, its prognostic value appears limited.

## 1. Introduction

About 75% of urothelial carcinomas of the urinary bladder manifest as non-muscle-invasive bladder cancer (NMIBC), while the remaining 25% are muscle-invasive bladder carcinomas (MIBC) [1,2]. The defining traits of NMIBC are its high recurrence rate necessitating repeated TURBTs and its tendency to progress, resulting in a relevant mortality risk, in particular for patients with high-risk NMIBC [3,4,5].

Intravesical therapy with Bacillus Calmette–Guérin (BCG) has proven in randomized controlled trials (RCTs) its efficacy in reducing recurrence and progression rates and is therefore the established gold standard for high-risk (HR) NMIBC patients opting for bladder preservation [2]. BCG therapy exerts its effects through a multifaceted immunological cascade. Upon internalization by urothelial cells via micropinocytosis, BCG triggers both direct cytotoxicity and an immunomodulatory response. This involves MHC II (major histocompatibility complex II) upregulation and cytokine secretion (e.g., IL-6, IL-8, TNF-α), culminating in the recruitment of various immune cells to the tumor microenvironment. The ensuing amplified cytokine response, featuring cytokines such as IL-1, IL-2, and IFN-γ, facilitates immune-mediated cytotoxicity, predominantly mediated by NK cells, CD8+ lymphocytes, and macrophages [6].

In addition to the immunotherapeutic approach of intravesical BCG, emerging research is assessing the value of immune checkpoint inhibitors (CPI) already within the NMIBC stage, either supplementing BCG in phase III trials or as a primary therapeutic agent in BCG-refractory patients in phase II trials [7]. Physiologically, the interaction between programmed death 1 (PD-1) as a receptor with its ligand programmed death ligand 1 (PD-L1) maintains immune homeostasis and prevents an overactive immune response [8]. PD-L1 may be expressed in this context by a variety of antigen-presenting immune cells, including macrophages, dendritic cells, and T-cells. In pathological contexts, however, numerous tumor types including bladder carcinoma overexpress PD-L1 in cancer cells, exploiting the PD-1/PD-L1 pathway for immune evasion [9]. Despite a reported general overexpression of PD-L1 in tumor cells compared to normal urothelium, the extent of PD-L1-expressing cells varies between individuals [10]. In BCG-resistant HR NMIBC, an increased number of PD-L1-expressing cells may be a sign of immune escape as well as a result of BCG therapy-mediated effects [11].

Several studies have additionally evaluated the predictive and prognostic significance of PD-L1 expression in NMIBC, demonstrating heterogeneous results. A study by Roumiguiè et al. was able to show a correlation between PD-L1 expression in tumor cells and disease-free survival (DFS) [12]. On the other hand, a recent study by Civriz et al. found no predictive value for PD-L1 expression in response to BCG treatment [13].

Given the established role of BCG in the treatment of high-risk NMIBC and the emerging significance of PD-L1 as both a prognostic marker and therapeutic target, the question arises as to how BCG therapy influences PD-L1 expression and what prognostic implication this has. Therefore, our study aims to investigate the hypothesis that BCG therapy significantly modulates the expression of PD-L1 on tumor cells and within the tumor microenvironment, correlating with clinical outcomes. For a comprehensive real-world analysis considering both tumor and tumor micro-environment (TME) immune cells, we applied the frequently used Combined Positivity Score (CPS), covering all PD-L1-expressing cells (tumor cells, macrophages, and lymphocytes, among others). Patients were dichotomized into positive (CPS > 10) and non-positive PD-L1 status groups (CPS ≤ 10), adhering to threshold values in RCTs [14]. To assess the temporal influence and the impact of an intravesical BCG therapy, two sequential tumor tissues from both BCG-treated and BCG-naïve patients were included in the analysis.

## 2. Materials and Methods

### 2.1. Patients

We conducted a retrospective analysis of patients diagnosed with high-risk (HR) NMIBC between 2010 and 2019 from our institutional database (single-center). To assess the variability of the PD-L1 expression at the individual patient level, our study criteria mandated the availability of a secondary TURBT specimen from the longitudinal follow-up. Further inclusion criteria were the classification of HR NMIBC based on the classification criteria of the EAU 2022 and/or the AUA 2020; patients qualified if they met the criteria of at least one of these classifications. Additional inclusion criteria included comprehensive data on clinically and histopathologically validated risk factors (age, sex, tumor status as primary vs. recurrent, tumor number, maximum tumor size, stage, concomitant CIS, and grade) obtained from the hospital database and post-TURBT histopathological reports. Histopathological staging was based on the AJCC (American Joint Committee on Cancer) TNM classification. Only tumors confirmed histopathologically across multiple intravesical sites were considered multilocular; a mere clinical description in the operative report was not considered sufficient for this criterion. Exclusion criteria encompassed patients with incomplete data, histologically confirmed variants, prior treatments (intravesical or systemic chemotherapies, radiation therapy, immunotherapies), other malignancies, or significant liver and kidney dysfunction (ALT/AST > 3× upper limit normal (ULN), bilirubin > 1.5× ULN, creatinine > 1.5× ULN) were excluded. The study was approved by the local ethics committee (ethics vote: 536/2021BO2).

### 2.2. Follow Up

In general, the follow-up was conducted in an out-patient setting according to the respective contemporary guideline of the German Society of Urology (S3 guideline). Data sources were internal hospital patient charts and external physician records; these were reviewed for time of recurrence and progression. Only histopathologically confirmed lesions were considered recurrences. Upstaging in pathologic T-stage and grading were considered progression in the second TURBT. For OS analysis, all deaths irrespective of their cause were counted as events. Patients still alive were censored at the date of last contact. 

### 2.3. Immunohistochemical Staining and Assessment

Histologic slides were stained by an automatic stainer (Ventana BenchMark ULTRA, Roche Diagnostics, Basel, Switzerland) using the DAKO clone 22C3-protocol associated with the ‘Combined Positivity Score’ (CPS) as assessment variable. This protocol has been established and is routinely used in association with Pembrolizumab treatment [15].

In brief, the CPS combines (=summarizes) the ‘tumor proportion score’ (TPS), which is the percental share of all membranous positively stained tumor cells among all tumor cells and a modified ‘immune cells’ (IC) score, that is, the share of positive tumor-associated immune cells (lymphocytes and macrophages) among all tumor cells [16]. This value multiplied with 100 reveals the numeric CPS score. Dilution of the primary antibody was 1:50. The used detection system was Detection system OptiView DAB detection kit (Ventana Medical Systems, Tucson, AZ, USA).

The evaluation was conducted in a blind manner by two independent reviewers; any divergent results were subjected to re-examination. The assessors comprised a student who had been intensively trained by a pathologist, and a certified biologist possessing extensive expertise in the analysis of uro-oncological histological specimens.

**Figure 1:**
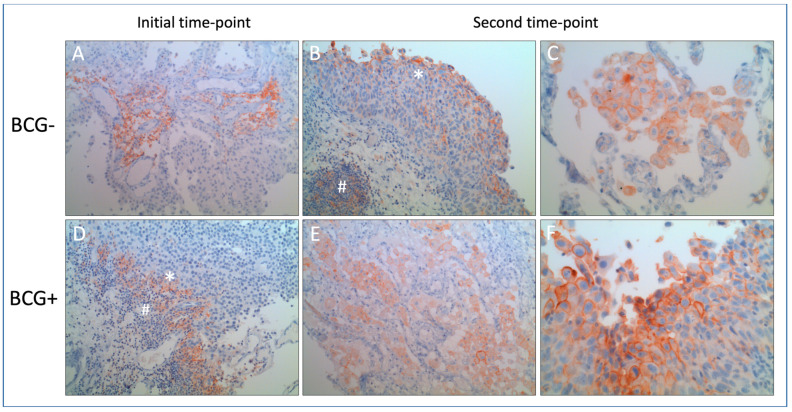
(**A**–**F**): Exemplary demonstration of tumor tissues’ PD-L1-expression at initial time-points (**A**,**D**) and follow-up time-points (**B**,**C**,**E**,**F**), in the groups without (upper) and with applied BCG (lower row). PD-L1 staining with DAKO clone 22C3 primary antibody and hematoxylin counterstaining. Magnification of 200× (**A**,**B**,**D**,**E**) and 400× (**C**,**F**). Membranous expression in tumor cells (*) and stained immune cells (#, lymphocytes, and macrophages considered for CPS) as brown staining. BCG = Bacillus Calmette–Guérin. CPS = combined positive score (see text).

Figure 1 shows exemplary staining results from both cohorts. Membranous expression in tumor cells and stained immune cells are visible as brown staining.

### 2.4. Statistical Analysis

CPS results were compared using the Wilcoxon matched-pairs signed rank test, chosen for its robustness to outliers, effectiveness for cohorts with limited sample sizes, and the absence of an assumed normal distribution. The correlation of CPS above median and positive initial PD-L1 status (defined as CPS > 10) as categorical variables with established clinicopathological parameters was analyzed using the Fisher’s exact test, chosen for its precision with categorical data in small sample sizes and its applicability regardless of expected value distribution across cells. Correlation analysis of CPS as continuous variable with established clinicopathological parameters was conducted using Spearman’s rank correlation analysis, allowing for the assessment of relationships without assuming a linear association. Kaplan–Meier analyses were performed to reveal data on TTR, PFS, and OS. Differences across subgroups were evaluated using the log-rank test. Univariate and multivariate testing for correlation of tested parameters with outcome were performed using Cox regression analyses. For multivariate analysis, various sets of parameters comprising BCG therapy, initial T-stage, CIS, grading, tumor size, multilocular lesions, age, sex, initial PD-L1 status positive, initial CPS > median, change of PD-L1 status, and change of CPS > median were investigated. To mitigate the risk of Type-I errors associated with multiple comparisons, a Bonferroni correction was implemented. *p* values < 0.05 were considered as statistically relevant. Statistical analysis and graphical illustrations were performed using GraphPad Prism 9 (GraphPad Software, La Jolla, CA, USA).

## 3. Results

An amount of 63 patients (56 male, 7 female) with an initial diagnosis of HR NMIBC and an additional available TURBT sample during follow-up were identified. A total of 126 tumor samples were therefore evaluated for their CPS and PD-L1 status (TURBT 1 and TURBT 2). The median age at initial diagnosis was 73.9 years (95% CI 69.70–78.50 years). Median follow-up was 77 months (range 11.3–140.5 months). An amount of 38 of the included patients received BCG (60.32%), and 25 patients did not receive BCG (39.68%). Reasons for this were a documented refusal by the patient, either because of comorbidities that spoke against BCG therapy (e.g., immunosuppression) or because of an increased age (median age BCG group 70.8 years, median age non-BCG group 80.9 years). Median tumor size was 2.2 cm (95% CI 1.7–2.7); multilocular tumors were present in 49 of the patients (77.78%). Survival follow-up and data on recurrence were available for 62 patients (98.41%). Table 1 summarizes the patient characteristics and clinicopathologic parameters of the patients. 

**Figure 2:**
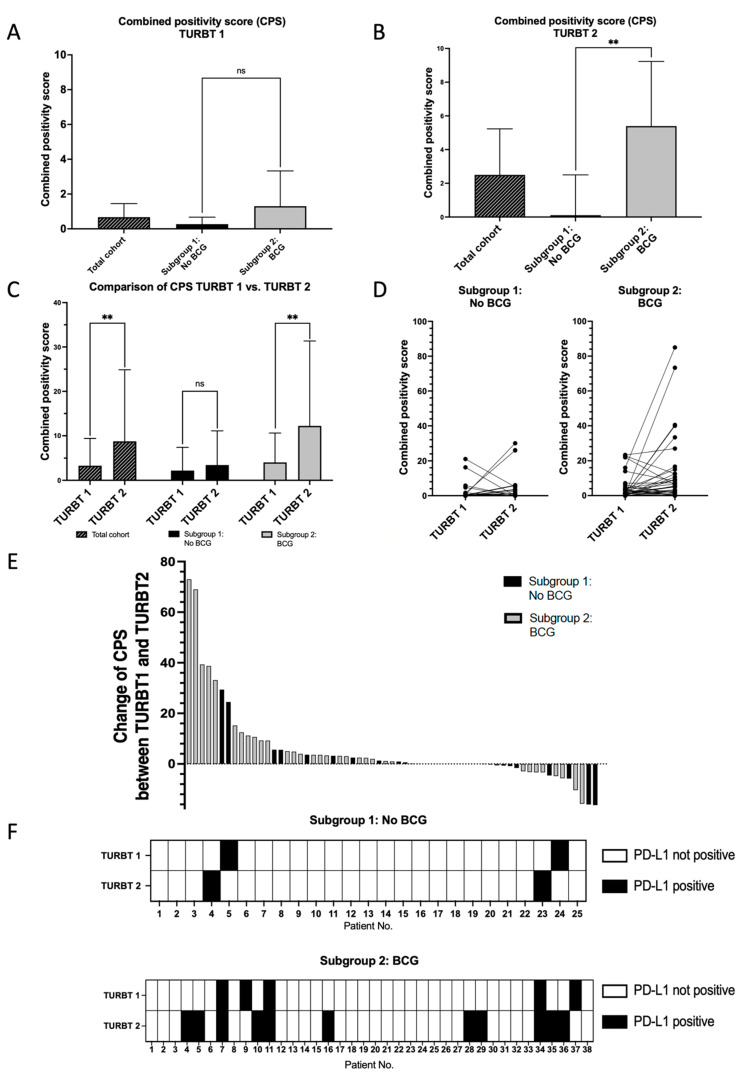
(**A**): Comparison of PD-L1 expression (Combined Positivity Scores, CPS) in tumor tissue from TURBT1 showed no significant difference between patients with and without BCG therapy in the subsequent course (median of entire cohort 0.67, no BCG vs. BCG 0.27 vs. 1.30, *p* = 0.1465). Legend: ns = not significant. (**B**): Comparison of CPS in TURBT2 tumor tissue shows a significantly higher CPS in BCG-treated patients compared to BCG-naïve patients (5.39 vs. 0.11, *p* = 0.0423). Legend: ** = statistically significant. (**C**): Comparison of CPS between TURBT1 and TURBT2 demonstrates a significant difference in the entire cohort based on the change of CPS in the BCG-treated group. Legend: ns = not significant; ** = statistically significant. (**D**): Change of individual CPS over time in BCG-naïve and BCG-treated group. (**E**): Waterfall plot of individual changes of CPS for each patient illustrates more distinctive changes in the BCG-treated group. (**F**): Illustration of PD-L1 status of individual patients in TURBT1 and TURBT2 shows an increase in the number of PD-L1 positive patients in the BCG-treated group.

The median CPS of the initial tumor samples was 0.67 (TURBT 1, 95% CI 0.17–1.45). The group subsequently receiving BCG did not show a significantly different CPS compared to the group not receiving BCG (1.30 vs. 0.27, *p* = 0.1465). At the initial time point, the observed CPS corresponded to a number of PD-L1 positive patients of seven (five in the BCG receiving group and two in the non-BCG receiving group). At the time of the second TURBT, the median CPS was 2.50 (TURBT 2, 95% CI 1.29–5.23), with the median CPS in the BCG group being significantly higher than the CPS in the BCG-naive group (5.39 vs. 0.11, *p* = 0.0423). A comparison of the evaluated time points within subgroups showed a significant difference comparing TURBT 1 and TURBT 2 in the BCG group (*p* = 0.0056), but not in the BCG-naive group (*p* = 0.5412). The median change in CPS in the overall cohort was 0.97 between both time points, showing more patients with an increase in CPS > Median in the BCG group than in the BCG-naïve group (23 vs. 8 patients). The median change of CPS was 2.78 in the BCG group and 0.00 in the BCG-naïve group (*p* = 0.075). The observed dynamics of the CPS magnitude accounted to an increase in PD-L1 positive patients from 5 to 11 in the BCG group and a constant number of two patients in the BCG-naïve group (however, they were different individuals). Figure 2 illustrates this data.

**Figure 3:**
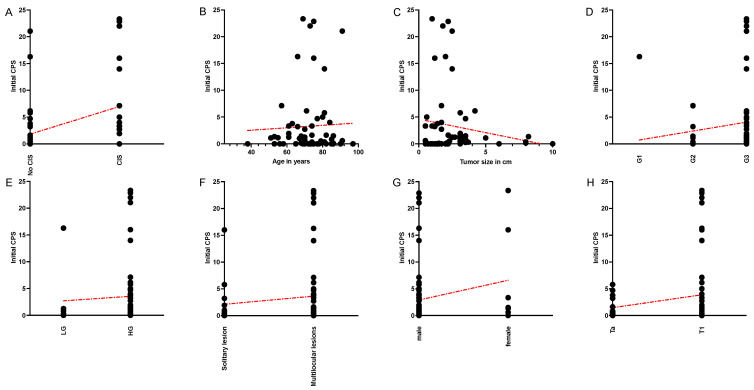
Graphical illustration of Spearman’s correlation of initial CPS and clinicopathological parameters ((**A**): CIS, significant; (**B**): Age, ns; (**C**): Tumor size, ns; (**D**): Grading G1–G3, significant; (**E**): Grading LG–HG, ns; (**F**): solitary vs. multilocular lesions, ns; (**G**): sex, ns; (**H**): Ta vs. T1, ns). Legend: Red line: Result of the linear regression analysis to illustrate the relationship between the respective variables.

The correlation analysis of an initial high CPS (CPS > Median) with established clinical and pathological risk factors (T1-stage, CIS, grading > LG/G1, tumor size > 3 cm, multilocular lesions, age > 70 years, male gender) showed no statistically significant correlations (Table 2). However, a positive PD-L1 status (defined as CPS > 10), correlated as statistically significant to the presence of CIS (*p* = 0.0169) and tumor size > 3 cm (*p* = 0.041). A correlation was found to be positive for CIS and negative for tumor size (OR 8.269 and 0.394, respectively). Spearman’s correlation analysis for continuous values of CPS showed a positive association of CIS and CPS (*p* = 0.0388) as well as grading G1–G3 (*p* = 0.0351) (Table 3 and Figure 3).

Survival analyses showed a median time-to-recurrence (TTR) of 14.03 months, a median progression-free survival (PFS) of 39.1 months, and a median overall survival (OS) of 114.5 months for the total cohort.

**Figure 4:**
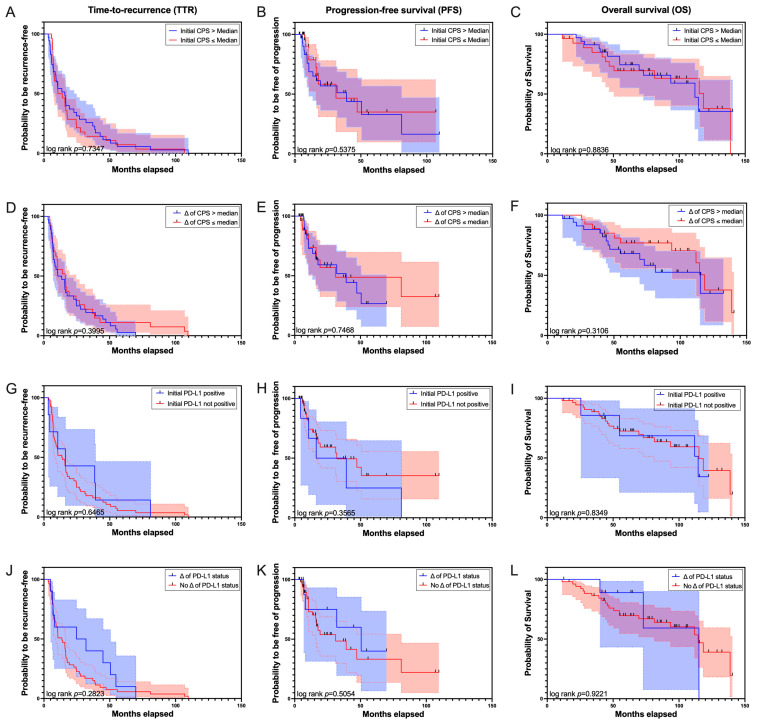
(**A**–**L**): Kaplan–Meier analyses don’t show significant differences in time-to-recurrence (TTR; (**A**,**D**,**G**,**J**)), progression-free survival (PFS; (**B**,**E**,**H**,**K**)) and overall survival (OS; (**C**,**F**,**I**,**L**)) based on initial CPS (**A**–**C**), distinct change of CPS (>Median, (**D**–**F**)), initial PD-L1 positivity (**G**–**I**) or status-change to PD-L1 positivity (**J**–**L**). Legend: Change of CPS > median, initial PD-L1 status positive and change from PD-L1 status negative to positive are illustrated in blue, 95% CI is illustrated as light blue shade between the dotted lines. Change of CPS ≤ median, initial PD-L1 status not positive, no change of PD-L1 status are illustrated in red, 95% CI is illustrated in light red shade between the dotted lines.

The prognostic relevance of an initial high CPS value (>median), an initial positive PD-L1 status, a distinct change in CPS values between TURBT 1 and TURBT 2 (change in CPS > median change), and a change in PD-L1 status between TURBT 1 and TURBT 2 was subsequently evaluated. None of the applied criteria showed prognostic value for TTR, PFS, or OS in this regard. Initial high CPS values showed a median TTR of 15.87 months (vs. 12.31 months; *p* = 0.7347), a median PFS of 39.1 months (vs. 31.15 months; *p* = 0.5375), and a median OS of 111.87 months (vs. 118.38 months for low values; *p* = 0.8836). A distinct change in CPS between both analyzed TURBTs revealed no significant prognostic effect either (TTR 12.09 vs. 16.26 months, *p* = 0.3995; PFS 39.1 vs. 31.15 months, *p* = 0.7468; OS: 115.12 vs. 118.38 months, *p* = 0.3106). An initial positive PD-L1 status showed a median TTR of 16.26 months (vs. 13.815 months with a not positive PD-L1 status, *p* = 0.6465) and a median PFS of 27.68 months (vs. 31.93 months, *p* = 0.3656 and a median OS of 114.5 months (vs. 115.12 months with not positive PD-L1 status, *p* = 0.8439)). The switch of PD-L1 status from not positive to positive also showed no prognostic relevance (TTR 28.32 vs. 13.6 months, *p* = 0.2823; PFS 50.53 vs. 31.15 months, *p* = 0.5054; OS: 115.12 vs. 114.5 months, *p* = 0.9221). Figure 4 illustrates the corresponding Kaplan–Meier (KM) curves.

The univariate Cox proportional hazard analysis neither indicated a statistically significant prognostic value for an initial CPS > median, a positive initial PD-L1 status, a distinctive change of CPS status (change to >median of change), or the change of an initially not positive PD-L1 status to a positive status. Similarly, the remaining parameters evaluated (BCG therapy taken, initial tumor stage T1, CIS present, grading > LG/G1, tumor size > 3 cm, multilocular lesion, age > 70 years, or male gender) did not show statistical significance (Table 4A).

In multivariate analysis, these latter factors were evaluated in combination with each of the first-mentioned factors (initial CPS > median, positive initial PD-L1 status, pronounced change in CPS status (change > median of change), and change of an initial not positive PD-L1 status to a positive status). This indicated a negative prognostic trend for an initial positive PD-L1 status for TTR, which, however, was not statistically significant (*p* = 0.0583, HR 3.392). In contrast, the change from a not positive to a positive PD-L1 status was statistically significant for a positive prognosis concerning TTR (*p* = 0.032, HR 0.4202). Furthermore, this parameter showed a non-significant trend regarding a positive prognostic significance for PFS (*p* = 0.0834, HR 0.3173) (Table 4B).

## 4. Discussion

In recent years, the significance of PD-L1 expression has markedly increased, particularly due to the breakthrough of checkpoint inhibitors in the treatment of various tumor types, including urothelial carcinoma. However, PD-L1 expression is variable and subject to a multitude of factors that complicate its evaluation and assessment. These include the subjectivity of immunohistochemical assessment, the antibodies used, varying cut-off values, the heterogeneity of expression in different tissue samples (tumor heterogeneity), and the effects of prior therapies [17,18,19]. For instance, a study by Deng et al. has demonstrated the upregulation of PD-L1 expression following radiation in a mouse model [20]. In the context of non-muscle-invasive bladder cancer (NMIBC), the current evidence remains limited, partially because of the yet-to-be-established role of systemic checkpoint inhibition in this stage. Limited research has also been conducted regarding the influenceability of PD-L1 expression by various intravesical instillation therapies such as mitomycin, gemcitabine, and doxorubicin, substances primarily used in intermediate-risk NMIBC. Given the elevated risk of developing metastatic disease and requiring systemic immunotherapy later on, our study focused on investigating the variability in PD-L1 expression, as determined by the Combined Positive Score (CPS), particularly in the context of intravesical Bacillus Calmette–Guérin (BCG) in patients with high-risk non-muscle-invasive bladder cancer (NMIBC) [21].

We found that the initial CPS for the entire cohort had a median value of 0.67; seven patients were initially PD-L1 positive showing a CPS > 10. A comparative analysis between subgroups receiving subsequent BCG therapy and those that did not revealed a significantly higher CPS in the BCG group in the second TURBT. Notably, individual CPS changes indicated more distinct differences in the BCG group compared to the BCG-naïve group. This corresponds to an increase from 5 to 11 PD-L1 positive patients in the BCG group, while the number of PD-L1 positive patients in the BCG-naïve group remained two (although they were different individuals).

Two possible reasons might explain the elevated CPS in the second TURBT: the immunogenic effect of BCG therapy influencing the composition of the tumor microenvironment (TME) including PD-L1-expressing immune cells and the immune-stress induced overexpression of PD-L1 as a mechanism to evade immune response [6,9]. However, even in the absence of BCG-induced immune stress, elevated PD-L1 expression might signify evasion from T-cell responses. Additionally, intrinsic signaling pathways, which may be influenced by epigenetic changes and genetic mutations, could also lead to increased PD-L1 expression [22]. Furthermore, cytokines might influence the tumor microenvironment, regardless of BCG exposure [6,23]. However, our study clearly indicates that CPS levels show significantly greater changes in patients treated with BCG. Our methodology could not conclusively determine if elevated CPS was primarily due to tumor cell-associated PD-L1 overexpression as an immune escape signal or a result of changes in the TME. Both of these mechanisms are profoundly impacted by BCG. Given the higher cellularity of TME due to increased immune cell activity, one might speculate that especially in cases of strong CPS shifts, TME alteration is the predominant mechanism; still, this remains unconfirmed.

Although BCG therapy’s effects on CPS and PD-L1 expression seem reasonable, current evidence in this context, particularly concerning NMIBC, is limited. A study by Hashizume et al., which observed a statistically significant increase in PD-L1 expression in urothelial tumor tissue post-BCG treatment in 22 BCG-resistant NMIBC patients, share similar conclusions but differ in methodology [11]. Unlike their study, which used an intensity score (IS) and proportion score (PS) similar to the Allred scoring system, we utilized a continuous CPS analysis for PD-L1 expression extended with a dichotomized categorical analysis using the clinically relevant and well-established cut-off value of 10 as the threshold for PD-L1 positivity. Inman et al. described in a 2007 paper the expression of PD-L1 in a cohort of 280 high-risk urothelial carcinoma patients (including advanced stages ≥ pT2) [24]. Based on the application of a divergent definition of PD-L1 positivity, the comparability to our results is again limited (PD-L1 positivity when ≥1% tumor cells with histologic membrane staining). Of note, however, is the high number of PD-L1 positive tumors in the presence of CIS (45% of CIS with PD-L1 positivity), which is in accordance with our observations (positive correlation of CIS and positive PD-L1 status). A subgroup analysis of 16 patients with recurrence after BCG therapy showed an extremely pronounced PD-L1 positivity in so-called BCG granulomata in 12/16 patients, potentially supporting the greater influence of TME for PD-L1.

Aydin et al. report, in their study in 141 patients with high-grade NMIBC, an initial PD-L1 positivity (defined as PD-L1 expression ≥ 1%) of 46.2%, which increases to a rate of 55.1% after BCG therapy [25]. All these studies, however, also underscore the challenges of comparing immunohistochemical studies due to differences in antibodies, assays, and cut-off values.

Contrary to these observations is the study by Bellmunt et al. [26]. Tumor samples from 160 patients with urothelial carcinoma were analyzed for PD-L1 expression (cut-off for PD-L1 positivity ≥ 5%). The authors could not show any association between prior BCG exposure and PD-L1 expression in tumor cells. However, only 17 patients received at least one instillation of BCG in the presented cohort; furthermore, no detailed pathological description of the included 23 NMIBC patients was provided.

PD-L1 expression analyses encompass several challenges necessitating careful consideration in the analyses of our data alongside that of the cited studies. Notably, significant variability exists in the immunohistochemical scoring among various pathologists [27]. Additionally, bladder cancer is characterized by one of the highest mutation rates among carcinomas, leading to substantial heterogeneity [28]. Such variability, a focal point of extensive research, hinders the establishment of broadly generalizable findings also for PD-L1 expression. In this context, the fluctuation of PD-L1 expression in longitudinal tumor development must also be considered: De Jong et al. demonstrated variability in PD-L1 expression in a matched analysis of TURBT samples, radical cystectomy specimens, and lymph node metastases [29]. Additionally, prior therapies might influence PD-L1 expression: A study by McDaniel et al. demonstrated a significant increase in PD-L1 expression in patients with urothelial carcinoma following neoadjuvant cytotoxic chemotherapy [30]. We aimed to minimize the impact of these variables in our study by excluding patients who had previously undergone systemic or intravesical chemotherapies of any kind. Moreover, the pathological assessment was consistently performed by the same duo, applying a dual-review principle.

We further determined the prognostic significance of CPS and PD-L1 status related to overall survival (OS), time-to-recurrence (TTR), and progression-free survival (PFS). While univariate and Kaplan–Meier analysis revealed no significant associations for initially high CPS, initially positive PD-L1 status, pronounced alteration of CPS and change of PD-L1 status from not positive to positive, and multivariate analysis including established clinicopathological parameters indicated potential prognostic implications for TTR and the change of the PD-L1 status. Similar, but non-significant trends were observed for a change of the PD-L1 status and PFS and for an initial positive PD-L1 status and PFS.

Various studies have reported inconsistent findings on the prognostic value of PD-L1 expression for NMIBC patient outcomes. Some authors describe a significant association of PD-L1 expression and survival; interestingly, immunohistochemical work suggests a negative prognostic value for OS, RFS, and CSS, whereas an mRNA-analyzing paper describes a positive prognostic value for CSS, RFS, and PFS [10,31,32]. A variety of other papers, by contrast, describe no prognostic significance, neither at the immunohistochemical nor at the mRNA level [22,33,34,35]. The study most comparable to our work shares our results and was not able to demonstrate an association of positive PD-L1 expression with recurrence-free survival (RFS) and PFS [25].

The discussed studies illustrate the challenges of comparing immunohistochemical retrospective studies. Differences in study collectives, assays, and antibodies on the one hand and varying criteria applied in the evaluation of histologic specimens on the other are major obstacles for reliable comparability. An exemplary illustration of this is a multicenter study by Roumigue et al., in which PD-L1 expression in tumor cells of 140 HR NMIBC is significantly associated with disease-free survival [12]. However, when applying antibody-specific cut-off values for categorization into PD-L1 positive and not positive, this significance disappeared. Furthermore, a study examining gene expression of, among others, PD-L1 in urine sediment over a longitudinal time course suggests a dynamic nature of PD-L1 expression peaking around the sixth treatment week and normalizing three months post-BCG therapy [36].

### 4.1. Limitations

Our study is subject to several limitations that must be considered when interpreting the results. Due to the real-world character of the cohort, it must be noted that the comparability of the study groups is limited due to their unbalanced nature. Additionally, the cohort size of 63 patients is relatively small. This is attributable to the nature of the study as a pilot project involving an authentic, real-life cohort in a university setting. Despite the use of statistical methods suitable for the data analysis of smaller samples (specifically, the Wilcoxon matched-pairs signed rank test and the Fisher exact test), which provide robust and valid results despite the challenge of small sample sizes, the limited statistical power of our analysis must be acknowledged, especially concerning the possibility of Type I and Type II errors. This applies in particular to the relatively low number of patients with an initial positive PD-L1 status and those experiencing a status change. Notably, the prognostic significance of a changing PD-L1 status for TTR is only evident in multivariate analysis, suggesting a possible mathematical effect rather than a genuine prognostic value. Therefore, the observed results must be prospectively validated in larger cohorts. Further limitations of our study are its single-center design and the inherent challenges of immunohistochemical analysis (potential bias by variations of tissue preparation and intraobserver variability). Additionally, the study’s retrospective framework necessitates the acknowledgement of inherent limitations such as potential selection bias, recall bias, and the potential for variability in the historical data’s quality and completeness. Although these factors can influence the interpretation of the results and limit the generalizability of the conclusions, the presented data substantially augments the existing literature, frequently relying on artificially implemented algorithms and cut-off values, in particular by using a well-defined cohort of HR NMIBC in association with clinically relevant histologic parameters (CPS with cut-off > 10).

### 4.2. Future Perspectives and Clinical Implications

While acknowledging its limitations, our investigation contributes valuable perspectives on the biological behavior of PD-L1 expression within NMIBC, particularly regarding ongoing clinical trials assessing the efficacy of CPIs in this setting. Our findings confirm the variable nature of PD-L1 expression in urothelial cancer and indicate its possible alteration by BCG. Assuming validation in larger cohorts, this might carry implications for treatment algorithms: despite all existing constraints, the PD-L1 status remains the best available and most widely used predictive biomarker for CPI response. Regarding the co-administration of CPI and BCG, our observation might provide insight into potential synergistic effects. Regarding the sequential administration of BCG and CPI, evaluating the predictive quality of PD-L1 expression after BCG therapy would be intriguing: are patients with increased PD-L1 post-BCG more likely to respond to subsequent CPI therapy and should patients without an increase directly be considered for more radical surgical interventions? These considerations are expected to be addressed within the framework of ongoing clinical trials and highlight the importance of understanding the biological behavior of PD-L1 expression. Our study offers preliminary insights based on retrospective observations which should be validated in prospective larger cohorts in the future.

## 5. Conclusions

In our investigation of high-risk non-muscle-invasive bladder cancer (NMIBC) tissue, we observed a significant elevation in the PD-L1 expression for patients post-BCG therapy. While the data regarding the prognostic value of the analyzed parameters should be interpreted with caution, the observed changes in CPS and PD-L1 suggest a potential for further analyses on the predictive power of post-BCG PD-L1 status in CPI therapy recipients.

## Figures and Tables

**Table 1:** Patients’ characteristics.

Initial TURBT		
	*n*	
Patients	63	100%
Male	56	88.89%
Female	7	11.11%
Age in years		
Median	73.9	95% CI: 69.70–78.50
Range	38.00–96.80	
Pathological stage		
pTis	18	28.57%
pTa	15	23.81%
pT1	30	47.62%
Grading		
WHO 1973		
G1	1	1.59%
G2	20	31.75%
G3	39	61.90%
X (unknown)	3	4.76%
WHO 2004/2016		
LG	7	11.11%
HG	53	84.13%
X (unknown)	3	4.76%
HR according to		
EAU 2022	61	96.83%
EAU 2020/AUA 2020	2	3.17%
BCG therapy		
yes	38	60.32%
no	25	39.68%
Variant histology		
Yes	0	0.00%
No	63	100.00%
Tumor size in cm		
Median	2.2	95% CI: 1.7–2.7
Range	0.5–10.0	
Multilocular lesions		
yes	49	77.78%
no	14	22.22%

Abbr.: TURBT = transurethral resection of bladder tumor; WHO = World Health Organization; LG = low grade; HG = high grade; HR = high risk; EAU = European Association of Urology; AUA = American Urological Association; BCG = Bacillus Calmette–Guérin.

**Table 2:** Correlation of high CPS levels (>Median) and PD-L1 positivity with clinicopathological parameters as categorical parameters reveals a significant association of PD-L1 positivity with CIS and tumor size > 3 cm. Statistically significant values in bold.

Parameter	Initial CPS > Median			Initial PD-L1 Status Positive		
	*p*-Value	Odds Ratio	95% CI	*p*-Value	Odds Ratio	95% CI
T1	0.453	0.6373	0.2529–1.764	0.429	0.4	0.07568–2.193
CIS	0.094	2.737	0.8954–9.234	**0.017**	**8.269**	**1.399–43.50**
>LG/G1	0.238	0.0013	0.0010–2.072	0.212	0.1091	0.005684–2.399
Tumor size > 3 cm	0.439	1.589	0.5827–4.395	**0.041**	**0.394**	**0.021–0.9339**
Multilocular lesions	0.365	2.035	0.6326–6.323	>0.9999	0.5513	0.04490–4.215
Age > 70 years	0.124	0.4174	0.1424–1.242	0.699	1.618	0.2935–8.633
Sex (male)	0.257	0.3467	0.06573–1.904	0.17	0.2451	0.04702–1.529

**Table 3:** The Spearman correlation, utilizing CPS as a continuous variable, indicates significant associations between CIS and CPS, along with grading (G1–G3) and CPS. Statistically significant values in bold.

Parameter	Initial CPS		
	Spearman’s *r*	95% CI	*p*-Value
CIS	**0.268**	**0.0139–0.4892**	**0.0338**
Age	−0.1105	−0.3553–0.1484	0.3884
Tumor size	0.0406	−0.2164–0.2924	0.7520
Grading G1–G3	**0.2727**	**0.0125–0.4983**	**0.0351**
Grading LG–HG	0.0716	−0.1931–0.3266	0.5867
Multilocular lesions	0.902	−0.1667–0.3388	0.4733
Sex (male vs. female)	0.1896	−0.0684–0.4239	0.1366
T1	0.041	−0.2163–0.2925	0.7513

**Table 4:** (**A**): Univariate Cox proportional hazard analyses for time-to-recurrence (TTR), progression-free survival (PFS) and overall survival (OS) do not show prognostic significance for the evaluated parameters. (**B**): Multivariate Cox proportional hazard analyses for various sets of parameters for time-to-recurrence (TTR), progression-free survival (PFS), and overall survival (OS) indicate a positive prognostic value of a status-change to PD-L1 positivity for the TTR.

(A)									
	TTR			PFS			OS		
	*p*	HR	95% CI	*p*	HR	95% CI	*p*	HR	95% CI
BCG therapy (yes/no)	0.8344	0.9464	0.5675–1.602	0.9203	1.042	0.4700–2.410	0.1068	0.5221	0.2337–1.157
Initial T-stage T1	0.1478	0.6878	0.4135–1.145	0.2045	0.5991	0.2678–1.330	0.3953	0.709	0.3189–1.588
CIS (yes/no)	0.1168	0.6279	0.3407–1.099	0.2263	0.563	0.2027–1.349	0.5704	0.779	0.3112–1.790
Grading > LG/G1 (yes/no)	0.2158	0.4067	0.06605–1.328	0.4967	0.494	0.02717–2.442	0.923	1.104	0.06141–5.337
Tumor size ≥ 3 cm	0.5749	0.8603	0.5121–1.474	0.7874	1.124	0.4937–2.776	0.4554	1.398	0.6046–3.617
Multilocular lesions	0.7691	1.094	0.6151–2.074	0.5642	1.338	0.5350–4.050	0.937	1.041	0.4152–3.168
Age > 70 years	0.8682	0.9573	0.5646–1.591	0.1223	0.4818	0.1742–1.149	0.1665	0.5185	0.1870–1.246
Sex (male)	0.5698	0.7943	0.3277–1.644	0.3381	0.4922	0.07875–1.677	0.3367	1.695	0.4920–4.491
Initial PD-L1 status positive	0.645	0.8297	0.3422–1.716	0.3712	1.573	0.5179–3.934	0.835	1.124	0.3204–3.049
Initial CPS > median	0.5698	0.7943	0.3277–1.644	0.3381	0.4922	0.07875–1.677	0.3367	1.695	0.4920–4.491
Change PD-L1 status (not positive to positive)	0.2838	0.6857	0.3247–1.309	0.5066	0.6906	0.1979–1.865	0.9222	0.9411	0.2212–2.757
Change CPS > median	0.3519	0.7562	0.4060–1.329	0.7839	1.127	0.4536–2.564	0.5782	0.7672	0.2758–1.849
**(B)**									
	**TTR**			**PFS**			**OS**		
	***p***	**HR**	**95% CI**	***p***	**HR**	**95% CI**	***p***	**HR**	**95% CI**
BCG therapy (yes/no)	0.9043	0.954	0.4391–2.050	0.2262	2.076	0.6302–6.884	0.1233	0.3373	0.07754–1.287
Initial T-stage T1	0.5632	0.8108	0.3895–1.627	0.848	0.8961	0.2720–2.646	0.1722	0.4354	0.1215–1.378
CIS (yes/no)	0.3408	0.6077	0.2191–1.720	0.1228	0.2637	0.04661–1.455	0.4708	1.972	0.3160–13.29
Grading > LG/G1 (yes/no)	0.0721	0.1897	0.02328–0.9600	0.3099	0.2633	0.01021–2.339	0.8214	0.7396	0.02844–7.214
Tumor size ≥ 3 cm	0.9431	0.977	0.5148–1.857	0.8426	0.8972	0.3065–2.674	0.1817	2.037	0.7268–6.058
Multilocular lesions	0.5697	1.21	0.6410–2.411	0.9131	1.062	0.3764–3.470	0.7069	1.245	0.4279–4.343
Age > 70 years	0.81	1.079	0.5770–1.992	0.1235	0.4089	0.1211–1.214	0.2767	0.5645	0.1852–1.500
Sex (male)	0.3855	0.6851	0.2689–1.521	0.313	0.4554	0.06937–1.728	0.3879	1.746	0.4424–5.927
Initial PD-L1 status positive	0.3848	1.532	0.5392–3.795	0.0583	3.392	0.8965–11.92	0.945	0.9514	0.2053–3.705
BCG therapy (yes/no)	0.8551	0.931	0.4288–2.002	0.3977	1.644	0.5143–5.285	0.1217	0.3365	0.07747–1.277
Initial T-stage T1	0.4804	0.7761	0.3753–1.547	0.5842	0.7349	0.2271–2.126	0.1728	0.436	0.1217–1.379
CIS (yes/no)	0.4273	0.6693	0.2512–1.841	0.2865	0.434	0.09389–2.115	0.4693	1.943	0.3323–12.64
Grading > LG/G1 (yes/no)	0.1054	0.2661	0.03828–1.080	0.6212	0.5628	0.02685–3.828	0.7716	0.7086	0.03312–5.113
Tumor size ≥ 3 cm	0.9512	1.02	0.5453–1.919	0.8697	1.089	0.3953–3.119	0.1809	2.026	0.7312–5.987
Multilocular lesions	0.6147	1.185	0.6260–2.369	0.9315	1.049	0.3739–3.438	0.7002	1.249	0.4347–4.344
Age > 70 years	0.8817	1.047	0.5654–1.908	0.1075	0.4124	0.1299–1.156	0.278	0.5652	0.1851–1.500
Sex (male)	0.3792	0.7284	0.3509–1.452	0.5791	0.528	0.02555–3.502	0.8762	1.05	0.5643–1.931
Initial CPS > median	0.4184	0.6997	0.2719–1.568	0.378	0.4951	0.07337–1.940	0.3766	1.723	0.4539–5.364
BCG therapy (yes/no)	0.6609	1.188	0.5433–2.555	0.1508	2.328	0.7177–7.426	0.123	0.3438	0.08057–1.277
Initial T-stage T1	0.36	0.7148	0.3405–1.444	0.4148	0.625	0.1876–1.847	0.1309	0.3871	0.1047–1.277
CIS (yes/no)	0.2257	0.5292	0.1908–1.508	0.163	0.326	0.06758–1.646	0.4671	1.954	0.3319–12.79
Grading > LG/G1 (yes/no)	0.0735	0.233	0.03370–0.9394	0.5587	0.5086	0.02450–3.412	0.7468	0.6828	0.03212–4.882
Tumor size ≥ 3 cm	0.9854	0.994	0.5203–1.909	0.9264	1.051	0.3659– 3.132	0.1828	2.02	0.7290–5.964
Multilocular lesions	0.2855	1.446	0.7514–2.934	0.6051	1.339	0.4677–4.467	0.5785	1.39	0.4659–4.933
Age > 70 years	0.8172	0.9295	0.4937–1.714	0.0643	0.3409	0.09937–1.000	0.2211	0.5111	0.1592–1.410
Sex (male)	0.7865	0.8871	0.3442–1.994	0.6909	0.7276	0.1076–2.892	0.2588	2.168	0.5067–7.974
Change PD-L1 status (not positive to positive)	0.032	0.4202	0.1819–0.9001	0.0834	0.3173	0.07624–1.080	0.4779	0.5773	0.1012–2.257
BCG therapy (yes/no)	0.8608	0.9335	0.4294–2.012	0.32	1.824	0.5555–6.082	0.1195	0.3363	0.07788–1.268
Initial T-stage T1	0.4755	0.7713	0.3694–1.550	0.5307	0.6989	0.2116–2.048	0.1631	0.4264	0.1184–1.354
CIS (yes/no)	0.4237	0.6649	0.2466–1.840	0.2189	0.3656	0.07289–1.883	0.4068	2.191	0.3539–14.98
Grading > LG/G1 (yes/no)	0.1225	0.2568	0.03382–1.207	0.447	0.3928	0.01731–3.119	0.9837	0.9734	0.03829–9.712
Tumor size ≥ 3 cm	0.9385	1.025	0.5450–1.952	0.751	1.188	0.4174–3.597	0.272	1.838	0.6358–5.764
Multilocular lesions	0.6114	1.188	0.6265–2.382	0.9354	1.047	0.3634–3.507	0.7443	1.206	0.4238–4.186
Age > 70 years	0.8674	1.054	0.5632–1.948	0.1451	0.4426	0.1378–1.272	0.2296	0.5196	0.1663–1.457
Sex (male)	0.4241	0.7021	0.2723–1.578	0.4793	0.5637	0.08222–2.297	0.4601	1.592	0.4112–5.160
Change CPS > median	0.9127	1.039	0.5089–2.008	0.3303	1.672	0.5686–4.642	0.6064	0.734	0.2083–2.279

## Data Availability

The data presented in this study are available upon request from the corresponding author. The data are not publicly accessible due to ethical constraints (Institutional review board statement).
